# Supplementary material for: Mutation‐Induced Pocket Deactivation: How Ser353/Pro245 Alters KCa2.2 vs. KCa3.1 Ligand Selectivity
Source: Arch Pharm (Weinheim). 2026 Jul 15;359(7):e70299. doi: 10.1002/ardp.70299 (PMC13373493; doi:10.1002/ardp.70299)
Supplement: Supplementary file 1 — Supporting File [file ARDP-359-e70299-s001.pdf]

SUPPORTING INFORMATION

Mutation-Induced Pocket Deactivation: How Ser353/Pro245 Alters $K_{Ca}2.2$ vs $K_{Ca}3.1$ Ligand Selectivity

Matteo Gozzi[1,2,#], Joana Massa[1,2,#,*], Oliver Koch[1,2,*]

[1] University of Münster, GRK 2515, Chemical biology of ion channels (Chembion), Corrensstraße 48, D-48149 Münster, Germany

[2] University of Münster, Institute of Pharmaceutical and Medicinal Chemistry, Corrensstraße 48, D-48149 Münster, Germany

[#] Shared First Authors

* Corresponding authors

Table of Contents

**Table S1**	Available $K_{Ca}2.2$ experimental structures	3
**Table S2**	Available $K_{Ca}3.1$ experimental structures	4
**Figure S1**	Sequence alignment of $K_{Ca}2.2$ and $K_{Ca}3.1$ channels across different species	5
**Table S3**	Average RMSD values obtained in the $K_{Ca}3.1$ MD simulations	6
**Figure S2**	$K_{Ca}3.1$ channel subunits RMSD values throughout the MD simulations	7
**Figure S3**	Calmodulin subunits RMSD values throughout the MD simulations	8
**Figure S4**	$K_{Ca}3.1$ channel Radius of gyration values throughout the MD simulations	9
**Figure S5**	Plots of the χ_1 and χ_2 dihedral angles of residue Trp322 during the $K_{Ca}2.2$ MD simulations	10
**Figure S6**	Trp216-Ser245 distances throughout the KCa3.1_open_P245S MD simulations	11
**Figure S7**	S5-Phelix-S6 Pocket calculated from the $K_{Ca}2.2$ and $K_{Ca}3.1$ channels	12
**Figure S8**	Results of the ensemble docking calculation of Compound 1	13
**Figure S9**	Results of the docking calculation of Compound 4 to the frame corresponding to the Trp216 *conformation a*	14
**Table S4**	Details of the molecular dynamics systems	15

Table S1: $K_{Ca}2.2$ structures published on the RCSB PDB database (https://www.rcsb.org/, accessed on 21.03.2026). For each structure the following details are indicated: PDB ID; species from which the $K_{Ca}2.2$ and calmodulin sequences were obtained; hydrophobic gate state (open/closed, based on distance between residue V390 in opposite subunits); co-determined ions; co-determine ligands (lipid molecules are excluded from this field); Trp322 orientation (defined as “Upward” if the side chain is directed towards the extracellular side, and “Downward” if the side chain is directed towards the intracellular side); mutations in the sequence; DOI of the reference paper.

* Ca2+ ions are bound to calmodulin C-Lobe but not to the N-Lobe.

PDB ID	Species	Determination Techniques	Resolution (Å)	Hydrophobic Gate State	Co-determined Ions	Co-determined Ligands	Trp322 Orientation	Sequence Mutation	Reference DOI
8V2G	Rattus norvegicus	Cryo-EM	3.18	Open	K^+, Ca^{2+}	/	Upward	/	10.1038/s41467-025-59061-1
8V2H	Rattus norvegicus	Cryo-EM	3.1	Closed	K^+, Ca^{2+}	AP14145	Upward	/	10.1038/s41467-025-59061-1
8V3G	Rattus norvegicus	Cryo-EM	3.1	Open	K^+, Ca^{2+}	UCL1684	Upward	/	10.1038/s41467-025-59061-1
9EIO	Rattus norvegicus	Cryo-EM	3.62	Open	K^*, Ca^{2+}	/	Downward	Mutation F244S	10.1038/s41467-025-59061-1
9O48	Homo sapiens	Cryo-EM	3.1	Open	K^+, Ca^{2+}	/	Upward	/	10.7554/eLife.107733
9O51	Homo sapiens	Cryo-EM	3.4	Closed	K^+, Ca^{2+} *	/	Upward	/	10.7554/eLife.107733
9O52	Homo sapiens	Cryo-EM	3.18	Closed	K^+, Ca^{2+}	Apamin	Upward	/	10.7554/eLife.107733
9O53	Homo sapiens	Cryo-EM	3.3	Closed	K^+, Ca^{2+}	Compound 1	Upward	/	10.7554/eLife.107733
9O5O	Homo sapiens	Cryo-EM	3.1	Open	K^+, Ca^{2+}	Compound 4	Upward	/	10.7554/eLife.107733
9O7S	Rattus norvegicus	Cryo-EM	2.71	Open	K^+, Ca^{2+}	NS309	Upward	/	10.1038/s41467-025-67232-3
9O85	Rattus norvegicus	Cryo-EM	3.13	Open	K^+, Ca^{2+}	Rimtuzalcap	Upward	/	10.1038/s41467-025-67232-3
9O93	Rattus norvegicus	Cryo-EM	2.96	Closed	K^+	Rimtuzalcap	Upward, flipped	/	10.1038/s41467-025-67232-3
9VU9	Homo sapiens	Cryo-EM	3.34	Closed	K^+	/	Upward	/	10.1038/s41467-026-68475-4
9VUA	Homo sapiens	Cryo-EM	3.23	Closed	K^+	AP30663	Upward	/	10.1038/s41467-026-68475-4
9VUB	Homo sapiens	Cryo-EM	3.35	Open	K^+, Ca^{2+}	Rimtuzalcap	Upward	/	10.1038/s41467-026-68475-4
9VUC	Homo sapiens	Cryo-EM	2.96	Closed	K^+	UCL1684	Upward		10.1038/s41467-026-68475-4

Table S2: $K_{Ca}3.1$ structures published on the RCSB PDB database (https://www.rcsb.org/, accessed on 21.03.2026). For each structure the following details are indicated: PDB ID; species from which the $K_{Ca}3.1$ and calmodulin sequences were obtained; hydrophobic gate state (open/closed, based on distance between residue V282 in opposite subunits); co-determined ions; co-determine ligands (lipid molecules are excluded from this field); Trp216 orientation (defined as "Upward" if the side chain is directed towards the extracellular side, and "Downward" if the side chain is directed towards the intracellular side); mutations in the sequence; DOI of the reference paper.

PDB ID	Species	Determination Techniques	Resolution (Å)	Hydrophobic Gate State	Co-determined Ions	Co-determined Ligands	Trp216 Orientation	Sequence Mutation	Reference DOI
6CNM	Homo sapiens	Cryo-EM	3.4	Closed	K^+	/	Downward	/	10.1126/science.aas9466
6CNN	Homo sapiens	Cryo-EM	3.5	Closed	K^+, Ca^{2+}	/	Downward	/	10.1126/science.aas9466
6CNO	Homo sapiens	Cryo-EM	4.7	Open	Ca^{2+}	/	Downward	/	10.1126/science.aas9466
9ED1	Homo sapiens, Rattus norvegicus	Cryo-EM	3.5	Closed	K^+, Ca^{2+}	DHP-103	Downward	/	10.1073/pnas.2425494122
9OA8	Homo sapiens, Rattus norvegicus	Cryo-EM	3.59	Open	K^+, Ca^{2+}	NS309	Downward	/	10.1038/s41467-025-67232-3
9YDZ	Homo sapiens	Cryo-EM	3.4	Closed	K^+	Rimtuzalcap	Downward	Mutation R355K	10.1038/s41467-025-67232-3
9Y5Q	Homo sapiens	Cryo-EM	4.73	Open	K^+, Ca^{2+}	Rimtuzalcap	Downward	Mutation R355K	10.1038/s41467-025-67232-3

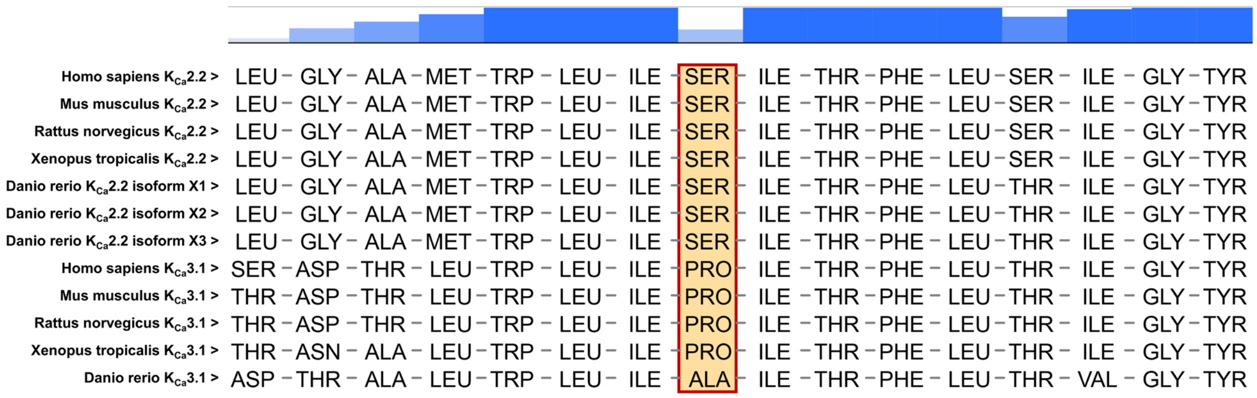

Figure S1: Multi sequence alignment (MSA) of K_{Ca}2.2 and K_{Ca}3.1 channel sequences across different species. Residues corresponding to S353 in the human K_{Ca}2.2 and P245 in the human K_{Ca}3.1 are highlighted. Sequences were retrieved from the Uniprot database (https://www.uniprot.org/), and correspond to the following Uniprot codes (from top to bottom in the figure): Q9H2S1, P58390, P70604, A0A6I8RA55, A0AC58GLV1, A0A8M2B2K1, A0AB32U3U6, O15554, O89109, Q9QYW1, A0A6I8QL32, X1WCL6. The sequence alignment was computed using the EMBL-EBI Clustal Omega web server (https://www.ebi.ac.uk/jdispatcher/msa/clustalo).

Table S3: Average RMSD values, and relative standard deviation, of the $K_{Ca}3.1$ channel subunits and the calmodulin subunits across the MD simulations. Trajectories were aligned on the Cα atoms of the $K_{Ca}3.1$ channel subunits, using the first frame of the production phase as a reference.

		RMSD ± sd (Å)	
**MD System**	**MD replica**	**$K_{Ca}3.1$ Channel Subunits**	**Calmodulin Subunits**
$K_{Ca}3.1$_closed_WT	Run 1	3.804 ± 0.402	5.315 ± 0.877
	Run 2	3.682 ± 0.213	5.254 ± 0.771
	Run 3	3.718 ± 0.330	5.652 ± 1.036
$K_{Ca}3.1$_closed_P245S	Run 1	3.749 ± 0.445	5.695 ± 0.741
	Run 2	3.634 ± 0.393	5.438 ± 0.649
	Run 3	3.586 ± 0.271	5.569 ± 0.660
$K_{Ca}3.1$_open_WT	Run 1	4.578 ± 0.490	4.469 ± 0.376
	Run 2	3.833 ± 0.284	4.608 ± 0.393
	Run 3	3.805 ± 0.275	4.330 ± 0.339
$K_{Ca}3.1$_open_P245S	Run 1	4.171 ± 0.409	4.304 ± 0.337
	Run 2	3.704 ± 0.317	4.166 ± 0.348
	Run 3	3.961 ± 0.253	4.400 ± 0.381

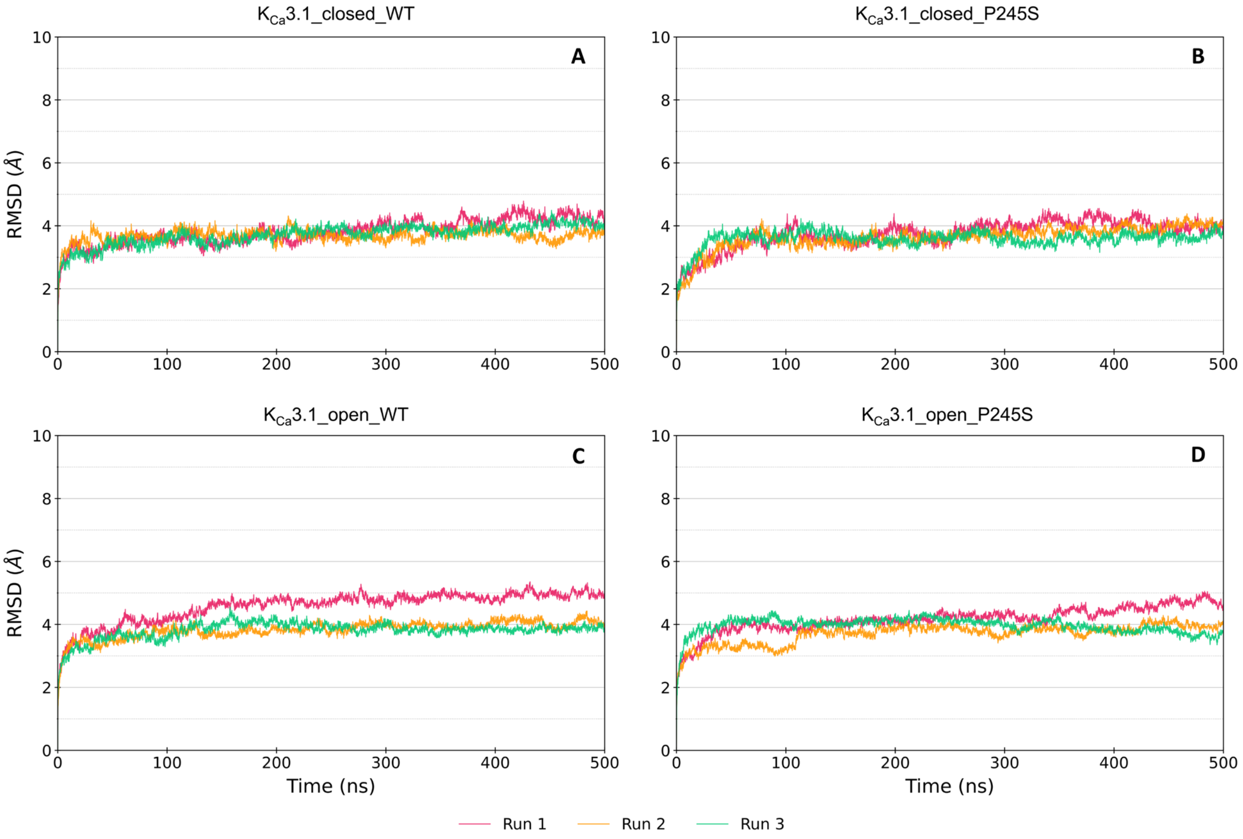

Figure S2: Root mean square deviation (RMSD) values calculated for the K_{Ca}3.1 channel Cα atoms across the triplicate MD simulations of the systems **A**) K_{Ca}3.1_closed_WT, **B**) K_{Ca}3.1_closed_P245S, **C**) K_{Ca}3.1_open_WT, and **D**) K_{Ca}3.1_open_P245S. Trajectories were aligned on the Cα atoms of the K_{Ca}3.1 channel subunits, using the first frame of the production phase as a reference.

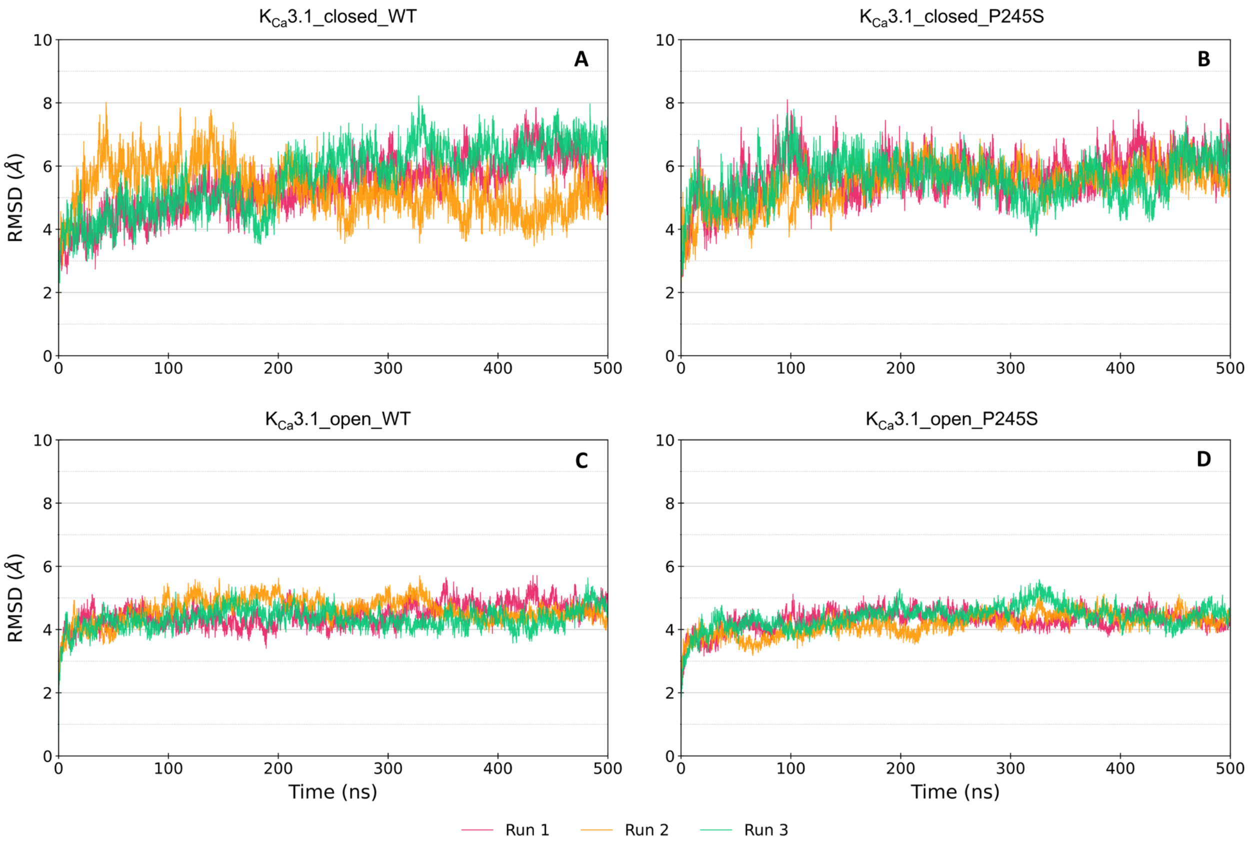

Figure S3: Root mean square deviation (RMSD) values calculated for the calmodulin subunits Cα atoms across the triplicate MD simulations of the systems **A**) K_{Ca}3.1_closed_WT, **B**) K_{Ca}3.1_closed_P245S, **C**) K_{Ca}3.1_open_WT, and **D**) K_{Ca}3.1_open_P245S. Trajectories were aligned on the Cα atoms of the K_{Ca}3.1 channel subunits, using the first frame of the production phase as a reference.

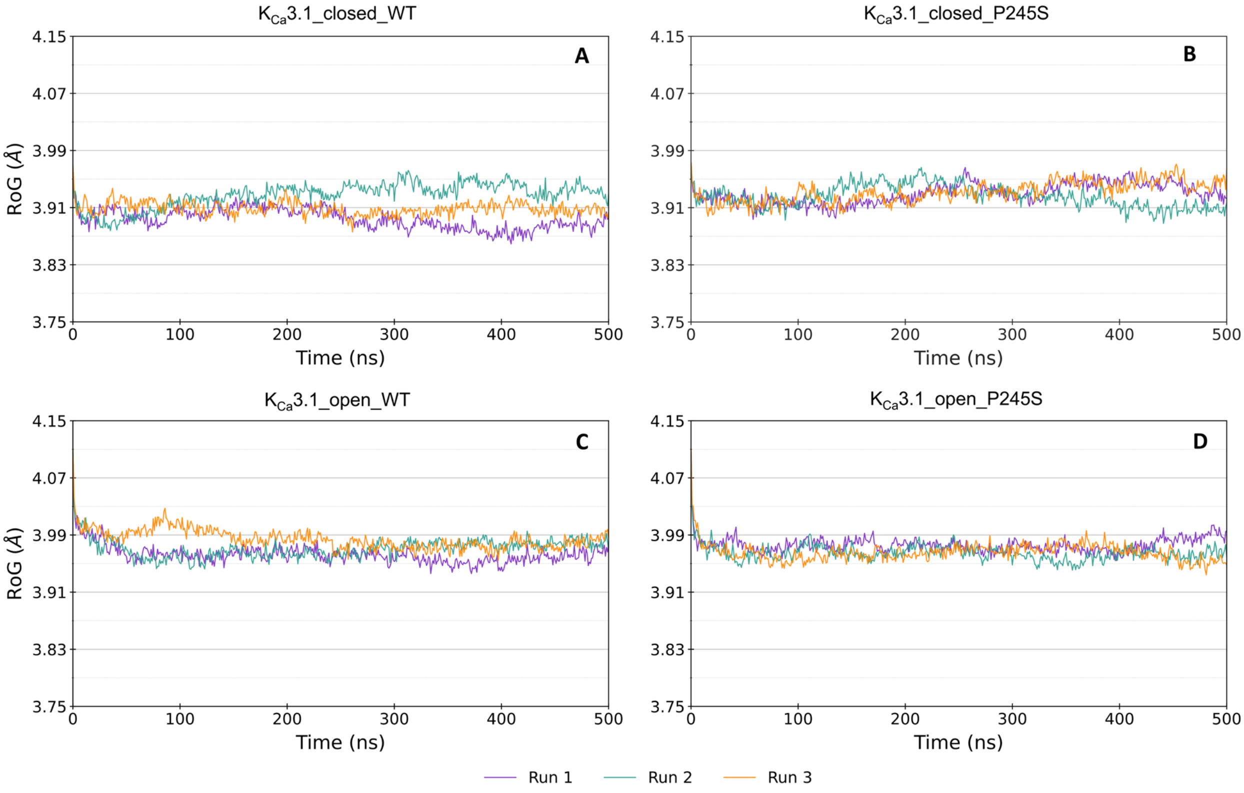

Figure S4: Radius of gyration (RoG) values calculated for the K_{Ca}3.1 channel subunits across the triplicate MD simulations of the systems **A**) K_{Ca}3.1_closed_WT, **B**) K_{Ca}3.1_closed_P245S, **C**) K_{Ca}3.1_open_WT, and **D**) K_{Ca}3.1_open_P245S. Trajectories were aligned on the Cα atoms of the K_{Ca}3.1 channel subunits, using the first frame of the production phase as a reference.

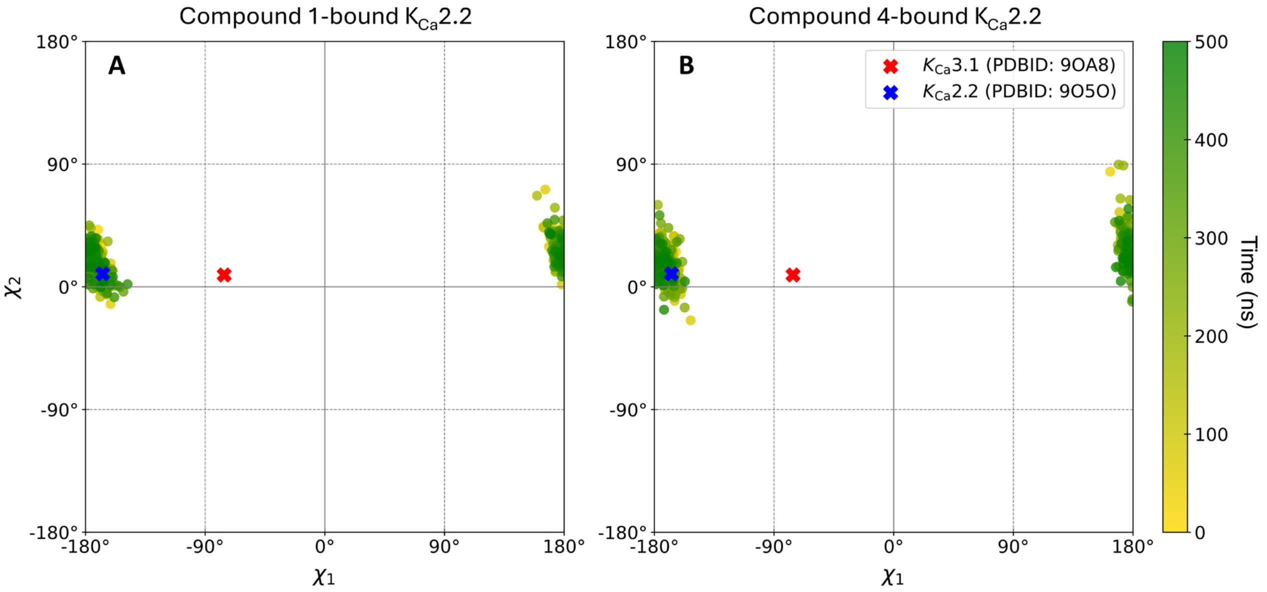

Figure S5: Plots of the χ_1 and χ_2 dihedral angles of residue W322 during the $K_{Ca}2.2$ MD simulations. Each dot represents a combination of dihedral angles in a specific frame of the trajectory. Time evolution along the simulation is represented by colour shift from yellow to green. Reference dihedral angles obtained from the Compound 4-bound $K_{Ca}2.2$ and $K_{Ca}3.1$_open cryo-EM structures are indicated with a blue and red cross, respectively. **A**) System Compound 1-bound $K_{Ca}2.2$, run 1, chain A. **B**) System Compound 4-bound $K_{Ca}2.2$, run 1, chain A.

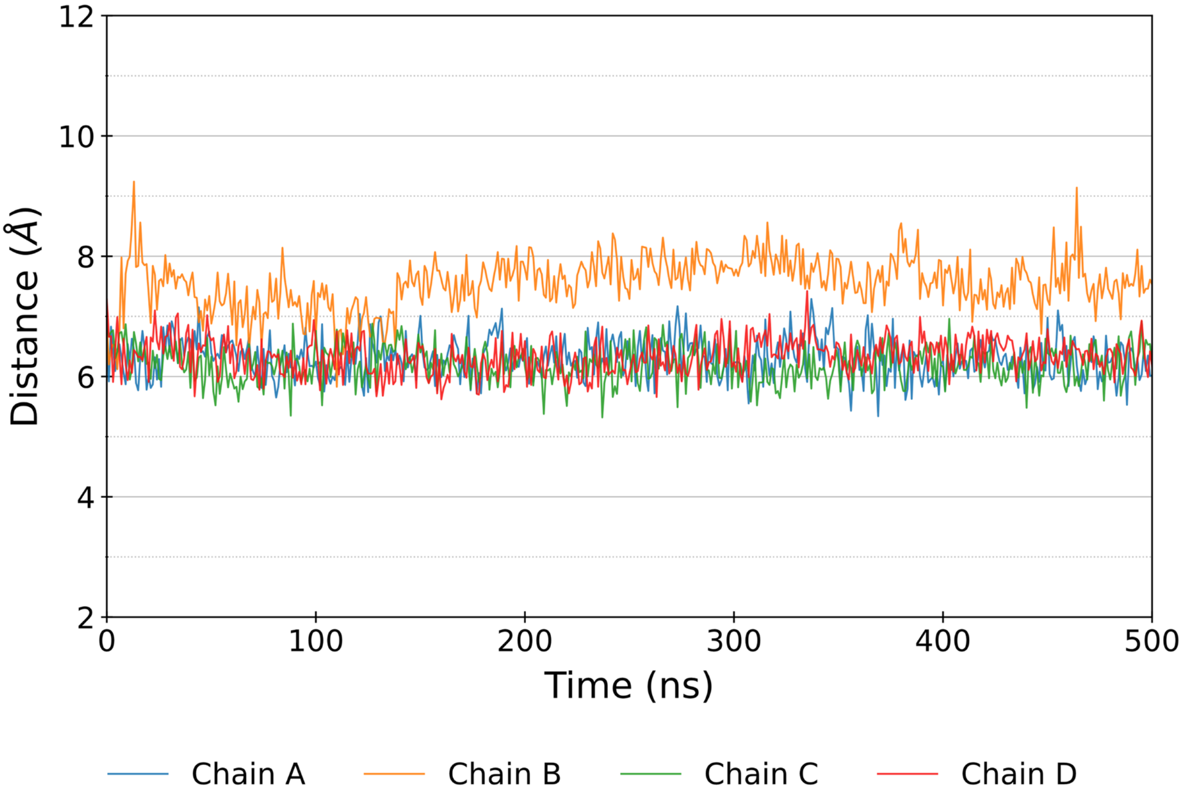

Figure S6: Distance between the W216 and the S245 Cα atoms in the distinct K_{Ca}3.1 chains, during the KCa3.1_open_P245S MD simulation, run2.

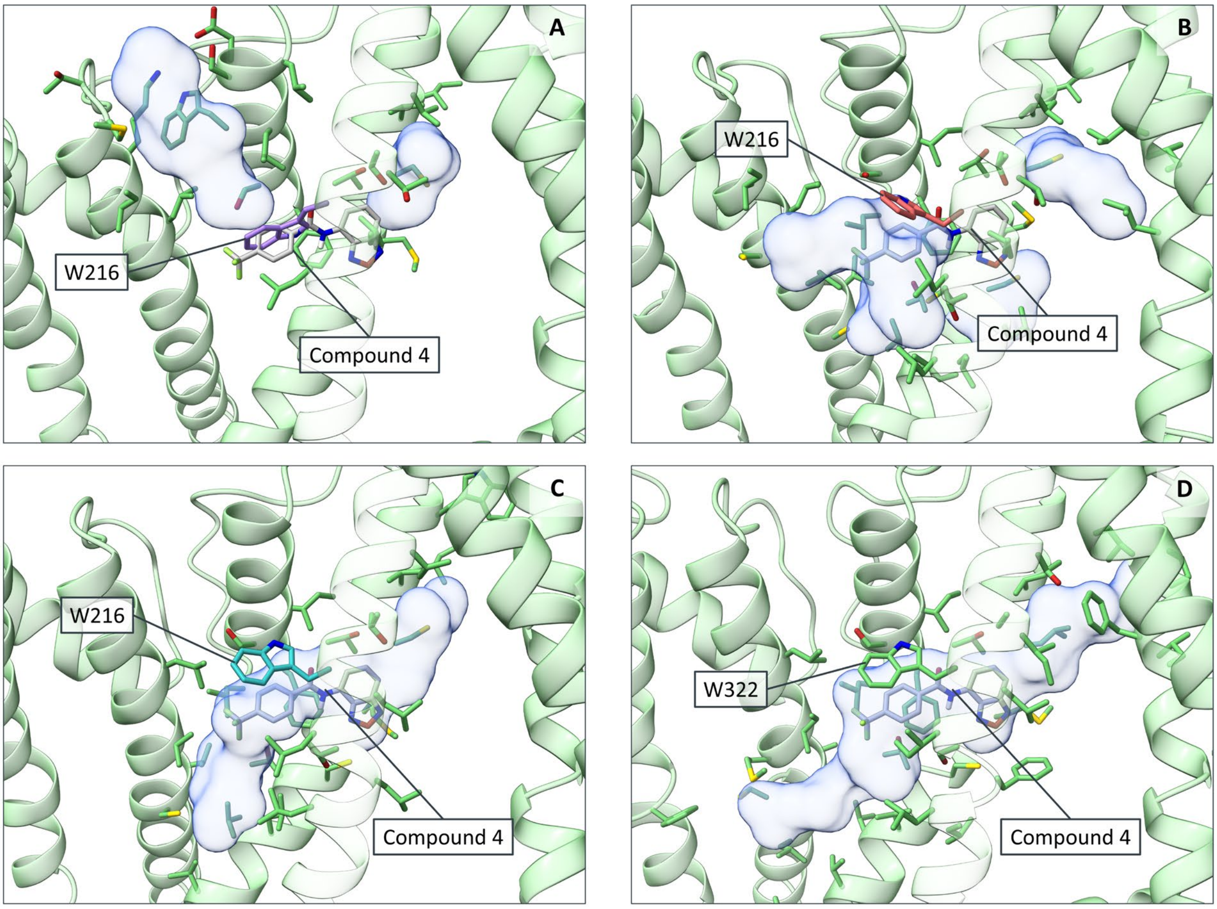

Figure S7: Pockets calculated with the fpocket software from the frames extracted from the $K_{Ca}3.1$ MD simulations and from the $K_{Ca}2.2$ structure. The cryo-EM binding mode of Compound 4 (PDB ID: 9O5O) is represented with grey sticks, as a reference. $K_{Ca}3.1$ and $K_{Ca}2.2$ channels are represented with green ribbons and sticks. The pockets calculated from the different channel conformations are represented with blue surfaces. The following structures are obtained from the $K_{Ca}3.1$_open_P245S simulation (run2), and represent **A**) Trp216 *conformation a*, **B**) Trp216 *conformation b*, and **C**) Trp216 *conformation c.* **D**) Compound 4-bound $K_{Ca}2.2$ cryo-EM structure (PDB ID: 9O5O). Only pockets within 5 Å from Compound 4 are included in the figure.

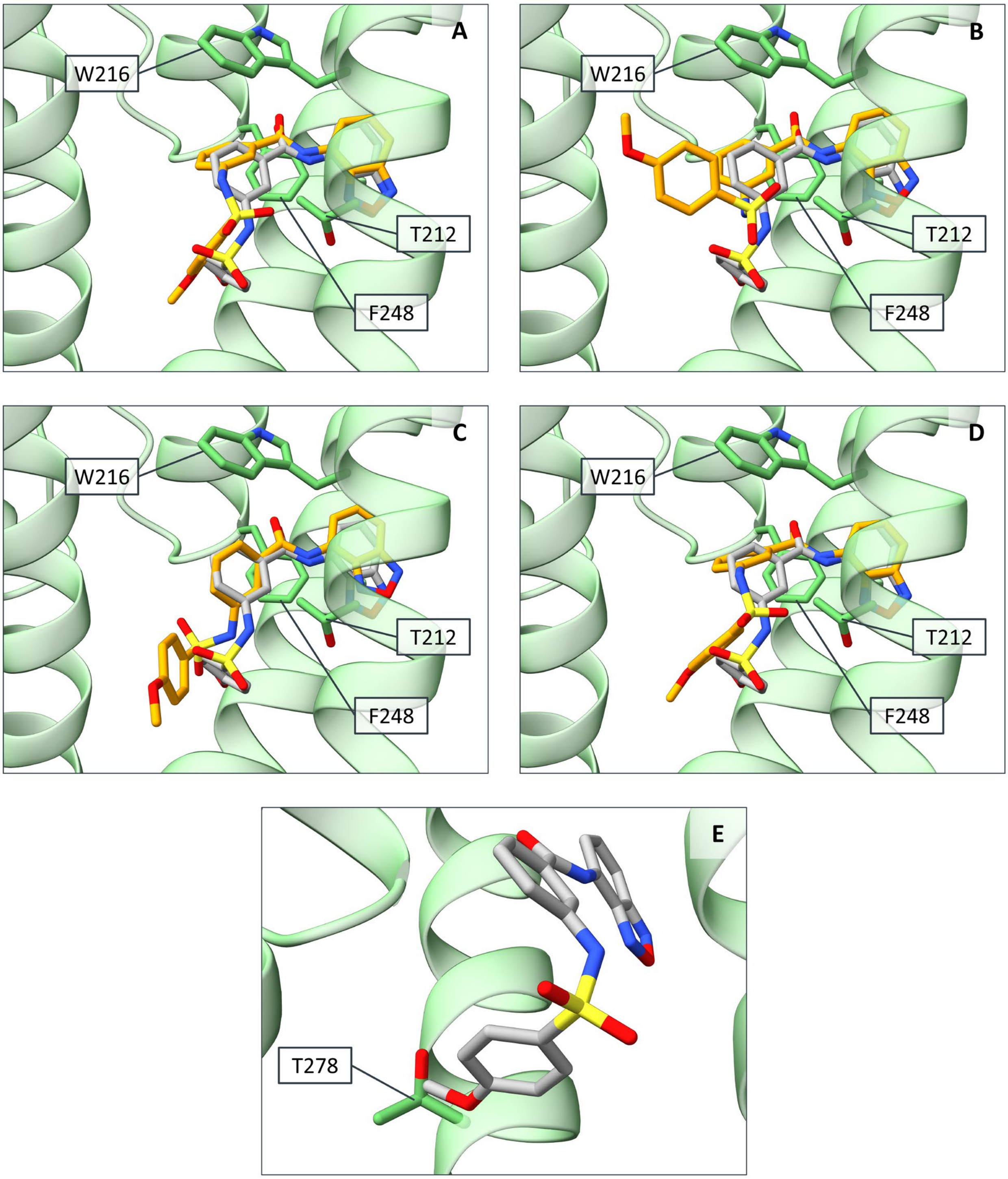

Figure S8: A-D) Results obtained from the ensemble docking of Compound 1 to the channel conformations extracted from the $K_{Ca}3.1$_open_P245S MD simulation. Only the results in which the benzoxadiazole/benzamide core adopted a conformation comparable to the cryo-EM structure are reported. **E**) Focus on the clash between residue T278 on the S6 helix of the $K_{Ca}3.1$ channel and the methoxybenzyl moiety of Compound 1. Due to this clash, the docking software is not able to dock the ligand with a conformation in line with the cryo-EM structure.

The $K_{Ca}3.1$ channel is represented by green ribbons, with residues in green sticks. Conformations obtained from the ensemble docking are represented with gold sticks, while the reference cryo-EM binding mode with grey sticks.

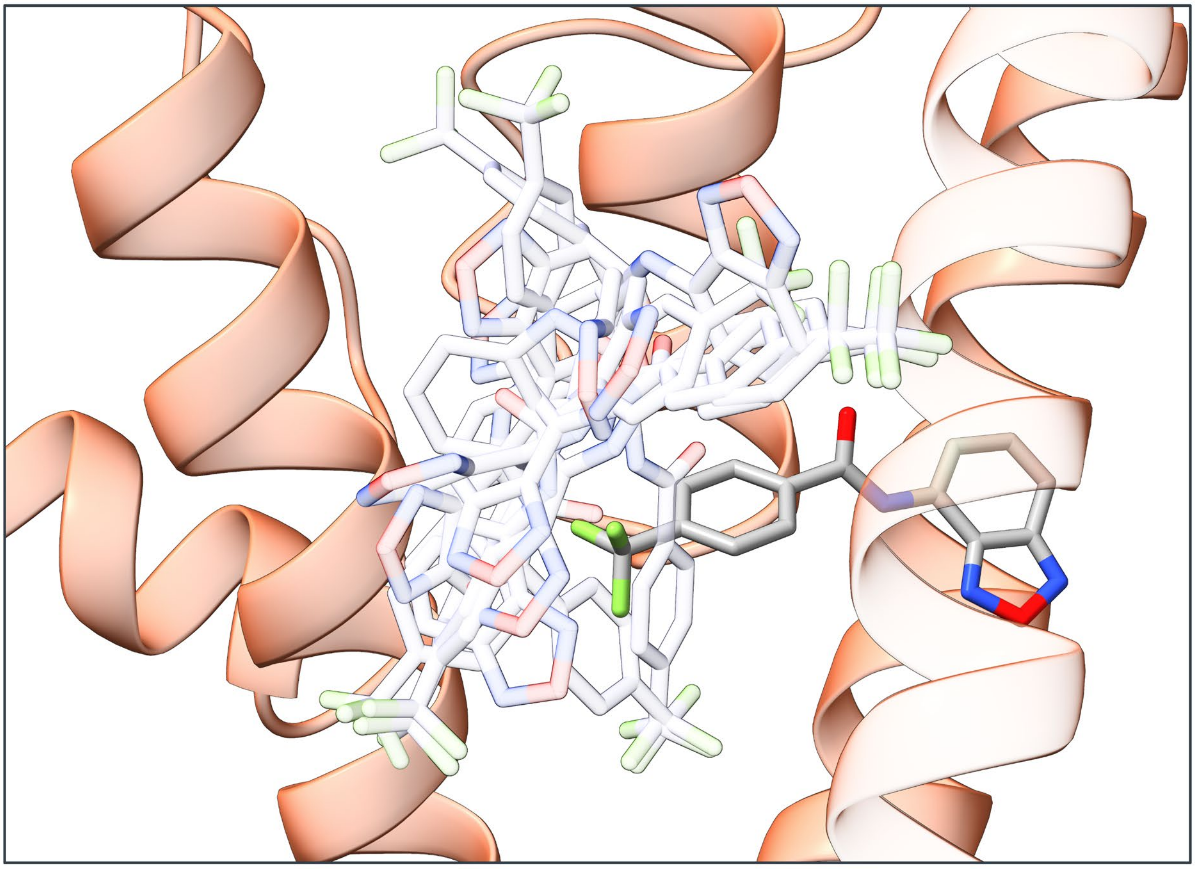

Figure S9: Docking poses obtained by docking Compound 4 to the conformation extracted from the $K_{Ca}3.1$_open_P245S MD simulation (run2, chain B) corresponding to 2 ns (W216 *conformation a*). $K_{Ca}3.1$ channel is represented with orange ribbons. The reference binding mode of compound 4 extracted from the Compound 4-bound $K_{Ca}2.2$ cryo-EM structure (PDB ID: 9O5O) is represented with grey sticks. Representative conformations of the 100 binding modes obtained through the docking calculation are represented with transparent light-blue sticks.

Table S4: Details of the MD systems $K_{Ca}3.1$_closed_WT, $K_{Ca}3.1$_closed_P245S, $K_{Ca}3.1$_open_WT, $K_{Ca}3.1$_open_P245S, Compound 1-bound $K_{Ca}2.2$, and Compound 4-bound $K_{Ca}2.2$. For each system, the following details are indicated: system name; initial dimensions of the box along the X, Y, and Z axis; number of POPC molecules contained in each membrane leaflet; number of K^+ and Cl^- ions added to the system; total number of water molecules; total number of atoms in each system.

	**Initial System Dimensions (Å)**			**N° POPC**		**N° Ions**			
**System Name**	**X**	**Y**	**Z**	**Outer Leaflet**	**Inner Leaflet**	**K^+**	**Cl^-**	**Water Molecules**	**Total Atoms**
$K_{Ca}3.1$_closed_WT	160.08	160.08	150.88	314	306	233	274	82,293	359,005
$K_{Ca}3.1$_closed_P245S	160.08	160.08	150.88	314	306	233	274	82,156	358,582
$K_{Ca}3.1$_open_WT	165.11	165.11	149.16	337	327	244	251	84,195	375,571
$K_{Ca}3.1$_open_P245S	165.1	165.1	149.25	337	327	244	251	84,278	375,808
Compound 1-bound $K_{Ca}2.2$	160.11	160.11	131.89	317	303	189	205	65,152	311,584
Compound 4-bound $K_{Ca}2.2$	160.12	160.12	130.38	320	302	186	200	64,273	309,077
